# Initial Screening of Poly(ethylene glycol) Amino Ligands for Affinity Purification of Plasmid DNA in Aqueous Two-Phase Systems

**DOI:** 10.3390/life11111138

**Published:** 2021-10-26

**Authors:** Nuno R. da Silva, Paula Jorge, José A. Martins, José A. Teixeira, João C. Marcos

**Affiliations:** 1 Centre of Biological Engineering, University of Minho, Campus de Gualtar, 4710-057 Braga, Portugal; paulajorge@ceb.uminho.pt (P.J.); jateixeira@deb.uminho.pt (J.A.T.); 2 Centre of Chemistry, University of Minho, Campus de Gualtar, 4710-057 Braga, Portugal; jmartins@quimica.uminho.pt

**Keywords:** aqueous two-phase systems, affinity partition, non-viral vectors, plasmid DNA purification, gene therapy, DNA vaccines

## Abstract

Gene therapy and DNA vaccination are among the most expected biotechnological and medical advances for the coming years. However, the lack of cost-effective large-scale production and purification of pharmaceutical-grade plasmid DNA (pDNA) still hampers their wide application. Downstream processing, which is mainly chromatography-based, of pDNA remains the key manufacturing step. Despite its high resolution, the scaling-up of chromatography is usually difficult and presents low capacity, resulting in low yields. Alternative methods that are based on aqueous two-phase systems (ATPSs) have been studied. Although higher yields may be obtained, its selectivity is often low. In this work, modified polymers based on poly(ethylene glycol) (PEG) derivatisation with amino groups (PEG–amine) or conjugation with positively charged amino acids (PEG–lysine, PEG–arginine, and PEG–histidine) were studied to increase the selectivity of PEG–dextran systems towards the partition of a model plasmid. A two-step strategy was employed to obtain suitable pure formulations of pDNA. In the first step, a PEG–dextran system with the addition of the affinity ligand was used with the recovery of the pDNA in the PEG-rich phase. Then, the pDNA was re-extracted to an ammonium-sulphate-rich phase in the second step. After removing the salt, this method yielded a purified preparation of pDNA without RNA and protein contamination.

## 1. Introduction

The increasing development of molecular biotechnology and molecular therapies, such as non-viral gene therapy and DNA vaccines, is reflected in an imperative demand for large amounts of plasmid DNA (pDNA) with a stringent clearance of impurities [1,2]. In both cases, pDNA plays a very important role as a non-viral vector. The use of this type of vector was extensively described for the expression of therapeutic proteins both in vitro and in vivo, making them important tools for gene therapy [3,4]. Moreover, pDNA vectors can stimulate humoral and cellular immune responses to a specific antigen, allowing for the development of DNA vaccination [5]. This was recently in the spotlight, with several DNA vaccines being developed against SARS-CoV-2 [6,7]. Although most of them are still in clinical trials, one plasmid-based vaccine for COVID-19 was recently approved in India [8]. In this regard, it is also worth mentioning that two DNA-based vaccines that use adenovirus as vectors (commercialised by Astra-Zeneca and Janssen) were already approved for clinical use in several countries around the world, including the European Union [9]. This opens good perspectives for the wide use of this vaccine strategy.

The wide application of pDNA requires adequate methods for its production and mostly for its large-scale purification. Consequently, the current biggest challenge consists in developing an efficient and cost-effective scalable purification process [10]. Currently, large-scale pDNA purification is mainly based on traditional chromatography methods, which provide final pDNA fractions that are separated from impurities, as well as undesired plasmid isoforms. Usually, ion exchange (IEC), hydrophobic interaction (HIC), and size-exclusion chromatography (SEC) are used, both in single or sequential mode, to meet all the regulatory requirements for pharmaceutical grade plasmids [10]. However, despite being widely used and the standard approach with an unmatched high resolution, chromatography-based techniques still have some disadvantages. These are mostly due to the long separation times and low capacity, which are related to problems with access and mass transfer within solid-phase chromatography matrices for large molecules, such as plasmids [11]. Furthermore, despite their high resolution, these techniques have high operational costs, require preliminary steps of purification, and their scale-up is frequently difficult to establish, which decreases the purification outcome [12,13]. Since large quantities of pDNA are needed for clinical use and due to its increasing number of applications, there is a need for more efficient and cost-effective processes for the production and downstream processing of pDNA of pharmaceutical grade [14].

Alternative downstream strategies have already been developed and implemented. On the one hand, new highly porous solid materials, known as monoliths, were tested to increase the capacity of the chromatographic systems. On the other hand, different separation methods, such as precipitation, extraction by organic solvents or aqueous two-phase systems (ATPSs), or even ultrafiltration, are widely implemented at the lab scale with relatively good yields and were tested for the large-scale purification of pDNA. Unfortunately, most of these alternatives are time-consuming, require hazardous chemicals or non-certified enzymes, neglect the regulatory guidelines, and are not scalable. Remarkably, ATPSs, which are a type of liquid–liquid extraction, have been used for the recovery and partial purification of a variety of biological products at different scales, making them a very promising alternative for pDNA purification [15,16]. ATPSs result from the mixture of two different polymers or a polymer and a salt in concentrations higher than critical values, forming two phases with different physical and chemical properties, which allows for the separation of components in a complex mixture [17]. This method presents advantages over the chromatography-based approaches, such as operational simplicity, easy scale-up, potential integration in a continuous process, low cost, capacity to integrate different process steps in one operation, and biocompatibility [18,19,20,21].

Although these systems are usually less selective when compared with the conventional methods, their selectivity may be improved by introducing a specific ligand to steer the biomolecule of interest into one of the phases [22]. In this approach, called affinity partition, the presence of affinity ligands, i.e., molecules with specificity and biorecognition properties towards a target solute, allows for the extraction of the target from a crude feedstock despite possible similarities with the contaminants [22].

ATPSs were already successfully used in a multitude of purification processes. These range from products for therapeutic purposes, such as antibodies, hormones, and enzymes [23,24,25,26,27,28,29,30,31,32,33], to others used in industrial applications, with emphasis on enzymes [34,35] and other proteins [36] employed in the food industry. In addition, they were also useful in recycling wastewater in the food, dairy, beverage, pharmaceuticals, dyeing, tannery, and metal-processing industries [37]. The implementation of ATPSs for pDNA purification was reported at different scales [38,39,40,41,42,43,44]. However, its utilisation is usually restricted to the first purification steps due to the low selectivity of the systems. The increase in selectivity was achieved by using specific ligands for pDNA. The cationic polymer polyethyleimine (PEI) derivatised with poly(ethylene glycol) (PEG) was used in PEG–dextran systems to selectively recover the pDNA in the polyplex form [45]. Although the polyplexes yield was very good (100%), attempts to separate them from the phase forming polymers via ultrafiltration resulted in adsorption to the membrane and very low final recovery. Alternatively, a protein-based ligand with glutathione-S-transferase protein (GST) fused to a zinc finger transcription factor (ZnF), designated GST-ZnF, was able to isolate pDNA with the ZnF recognition site in a PEG–dextran ATPS [46]. However, the pDNA was not eluted from the complex. The DNA-binding fusion protein LacI–His6–GFP, together with the conjugate PEG–IDA–Cu(II), was tested as an affinity ligand in PEG–dextran ATPSs [47]. Similarly, the elution of the pDNA was found to be the critical step and only 27% of plasmid recovery was achieved. Recently, the performance of a synthetic oligonucleotide that was employed as affinity ligand towards the plasmid vector pUC118 was described in PEG–sodium sulphate systems [48]. Although significantly good results were reported, namely 67% pDNA recovery, this approach is highly dependent on the nucleotide sequences of both elements to promote the recognition.

Interactions of proteins with nucleic acids are ubiquitous in nature, as they are the base of many fundamental biochemistry processes, such as DNA replication, transcription, and translation. These interactions are mediated by the amino acids that are present in the proteins and involve van der Waals interactions and several hydrogen bonds and water-mediated bonds [49]. Positively charged amino acids, such as lysine and arginine, mediate most interactions between proteins and nucleic acids, with interactions with guanine being overrepresented within all the amino acids groups [50]. The strong interaction of positively charged amino acids, such as lysine, arginine, and histidine, with nucleic acids was already explored in chromatographic separations of these biomolecules. The amino acids were used as affinity ligands that were bound to agarose supports for the purification of both pDNA and RNA [51,52]. The obtained results were very promising and indicate that these amino acids are good ligands for pDNA purification [51,53,54,55,56,57,58,59]. Purification processes were reported with yields of 45% for lysine and histidine and 79% for arginine. For all ligands tested, pDNA was recovered from the matrix without contaminants (more than 97% purity) [53,55].

Taking this into account, the main goal of this work was to screen four different PEG–amino affinity ligands to attempt to increase the selectivity of PEG 600 and dextran 100 ATPSs by adding affinity ligands based on the derivatisation of PEG with amino groups (PEG–amine) or conjugated with positively charged amino acids (PEG–lysine, PEG–arginine, and PEG–histidine) towards pDNA.

## 2. Materials and Methods

### 2.1. Materials

The model plasmid used was pVAX1/LacZ type ColE1 with 6050 bp, which was designed by Invitrogen (Carlsbad, CA, USA) for the development of DNA vaccines. *Escherichia coli* DH5α from Invitrogen was used as the host for this plasmid. Genetically modified *E. coli* DH5α (ackA-pta) (poxB), transformed with the NTC7482-41H-HA plasmid (6212 bp), were provided courtesy of the Nature Technology Corporation–Biologics by Design (Lincoln, NE, USA). PEG MW 600 (PEG 600), dextran 100,000 g·mol^−1^ (dextran 100), and methoxypolyethylene glycol amine 5000 (PEG–amine) were obtained from Sigma-Aldrich (St. Louis, MO, USA). Lysine, arginine, and histidine were obtained from BDH Chemicals (Poole, UK). All the other reagents used were of analytical grade.

### 2.2. Poly(Ethylene Glycol)–Amine Affinity Ligands Synthesis

#### 2.2.1. Methoxypolyethyleneglycol–Lysine and –Arginine

To an ice-cooled solution of PEG–amine in dichloromethane, Boc-Lys(Boc)-OH (or Boc-Arg(Pbf)-OH), hydroxybenzotriazol (HOBt), and dicyclohexylcarbodiimide (DCC) were sequentially added. The reaction mixtures were stirred in an ice bath for 2 h, allowed to reach room temperature (around 23 °C), and further stirred overnight. The precipitated dicyclohexylurea (DCU) was filtered off and both dichloromethane solutions were concentrated under reduced pressure. The slow addition of diethyl ether to the stirring dichloromethane solutions gave rise to copious white solids. The precipitates were collected via filtration, redissolved in dichloromethane, and precipitated again by adding diethyl ether. This procedure was repeated twice. The white solids were dried at room temperature overnight, dissolved in hydrochloric acid, and the solutions were left stirring at room temperature overnight. The solvent was removed at reduced pressure at room temperature to afford a light yellow vitreous solid. The solids were dissolved in dichloromethane and precipitated under stirring by adding diethyl ether. The final precipitates (PEG–lysine and PEG–arginine) were filtered off and dried at room temperature. The derivatisation of the polymers was confirmed using ^1^H NMR spectroscopy.

#### 2.2.2. Methoxypolyethyleneglycol–Histidine

To an ice-cooled solution of PEG–amine in dichloromethane, 1.2 equivalents of diciclocarbodiimide, 1.2 equivalents of hydroxibenzotriazol, and 1.2 equivalents of Boc-histidine were sequentially added. The reaction mixture was stirred for 16 h and then filtered and totally dried under reduced pressure. The precipitate was collected, redissolved in ethyl acetate, and extracted with potassium bisulphate (step repeated three times). The aqueous phase was acidified up to pH 7.0 and the final compound was extracted with dichloromethane (step repeated three times). The organic phase was completely evaporated, PEG–histidine was dissolved in dichloromethane and trifluoroacetic acid, and the reaction mixture was stirred for 16 h. In the end, the solution was evaporated under reduced pressure, the compound precipitated with petroleum ether, and finally filtered. The derivatisation of the polymer was confirmed using ^1^H NMR spectroscopy.

### 2.3. Plasmid Production

*E. coli* DH5α, previously transformed with plasmid pVAX1/lacZ, was grown in Luria-Bertani (LB) medium (yeast extract 0.5% (*w*/*v*), triptone 1% (*w*/*v*), and sodium chloride 0.5% (*w*/*v*), pH 7.4) with 30 μg·mL^−1^ of kanamycin, obtained from Sigma-Aldrich (St. Louis, MO, USA). The cultures were incubated at 37 °C and 180 rpm, overnight. The cells were harvested via centrifugation (4500× *g*, 10 min, 4 °C) at the end of the exponential phase and then stored at −20 °C.

### 2.4. Alkaline Cell Lysis

The cell lysate was prepared as described by Ribeiro et al. [39] (slightly modified, as reported by Sambrook [60]). Briefly, a bacterial pellet corresponding to 250 mL of bacterial culture was resuspended in 12.5 mL of a solution containing 50 mM glucose, 25 mM Tris-HCl, and 10 mM EDTA, pH 8.0. The suspension was gently stirred for 10 min in an ice bath while slowly adding 12.5 mL of a solution of 1% (*w*/*v*) SDS and 200 mM sodium hydroxide for the lysis to occur. Then, the solution was neutralised with 9.4 mL of a solution containing 3 M potassium acetate and 11.5% (*v*/*v*) acetic acid. The final mixture was centrifuged at 15,000× *g* for 20 min at 4 °C. The obtained supernatant, named lysate, was stored at −20 °C.

### 2.5. Lysate Desalting

A PD-10 Desalting Column from GE Healthcare Biosciences (Pittsburgh, PA, USA) was used for the cell lysate desalinisation. The column was first equilibrated with 25 mL of Tris-HCl (50 mM, pH 7.5 or 8.5). Then, 2.5 mL of the alkaline lysate added to the column was eluted with 3.5 mL of the same buffer, resulting in the collection of 3 mL of desalted lysate. The desalted lysate was stored at −20 °C.

### 2.6. Aqueous Two-Phase Experiments

Polymer–polymer ATPSs composed of 16.2% (*w*/*w*) PEG 600 and 17.4% (*w*/*w*) dextran 100 were prepared by mixing suitable amounts of each component’s stock solution. A total of 20% (*w*/*w*) of desalted *E. coli* lysate was added by considering the total system’s weight. Increasing amounts of PEG–amino affinity ligand were added and the desired total weight of the systems was adjusted with water. The components were mixed using tube inversion and vortexed. To accelerate the two phases’ settling, the mixtures were then centrifuged at 3000× *g* for 30 min at 25 °C.

### 2.7. Extraction with an Ammonium-Sulphate-Rich Phase

The top phases of the previously prepared 16.2% (*w*/*w*) PEG 600–17.4% (*w*/*w*) dextran 100 systems were recovered and then mixed with a new phase containing ammonium sulphate. For this purpose, the necessary amounts of salt and PEG 600 were calculated to obtain a new system with a final composition of 20% (*w*/*w*) PEG 600 and 15% (*w*/*w*) ammonium sulphate. This new system was used by Trindade et al. [40], yielding most of the pDNA in the bottom phase. The calculations were based on the addition of a lower phase with the same weight as the recovered upper phase. The partitioning experiments with the new systems were performed as previously described.

### 2.8. Nucleic Acids Partitioning Analysis by Agarose Gel Electrophoresis (AGE)

Samples from the top and bottom phases were analysed in 1% (*w*/*v*) agarose gels in the presence of ethidium bromide (EtBr). The loaded samples (10 µL) were prepared by mixing 2 µL of the loading solution buffer containing bromophenol and glycerol to 10 µL of the phase sample. The gels were run for 55 min in 90 V and 60 A in the presence of a TAE buffer (40 mM Tris-base, 20 mM acetic acid, and 1 mM EDTA, pH 8.0). They were then photographed using the gel documentation software Quantity One 1-D analysis from Bio-Rad (Hercules, CA, USA).

### 2.9. Total Protein Partitioning Analysis Using Polyacrylamide Denaturing Gel Electrophoresis (SDS–PAGE)

Samples of each ATPSs’ phase were evaluated in polyacrylamide denaturing gels (4% (*w*/*v*) stacking gels with Tris-HCl 0.5 M, pH 6.8, and 10% (*w*/*v*) resolving gels with 1.5 M Tris-HCl, pH 8.8). Samples were mixed with the sample buffer (0.5 M Tris-HCl, pH 6.8, 10% (*w*/*v*) SDS, 0.5% (*w*/*v*) bromophenol blue, and glycerol) and heated in a boiling water bath (95 °C) for 4 min to achieve the total denaturation before loading. The gels were run for 40 to 50 min at 170 V and 50 mA in the presence of the running buffer (3.03% (*w*/*v*) Tris, 14.4% (*w*/*v*) glycine, and 1% (*w*/*v*) SDS) [61,62]. Silver staining was used to visualise the protein bands in the gels [63,64].

### 2.10. Total Protein Quantification

The total protein content present in each of the ATPSs’ phases was quantified using the Bradford method, as described by Bradford [65]. The samples were read against blanks, which were prepared as follows. For the blank correspondent to the lysate, a mixture of the buffers used in its preparation was created with the same final composition, excluding the bacterial cells. Then, bottom and top blank samples were obtained by preparing ATPSs with the same composition but replacing the cell lysate with the previous blank mixture. Concentrations were determined from a calibration curve using bovine serum albumin (BSA, Sigma) as the standard.

### 2.11. Densitometric Analysis of Agarose Gel Bands

ImageJ 1.8 software (U.S. National Institutes of Health, Bethesda, MD, USA) [66,67] was used to compare the intensities of the pDNA and RNA bands on the agarose gels, where the top and bottom phases of the second ATPS (20% (*w*/*w*) PEG 600 and 15% (*w*/*w*) ammonium sulphate were analysed. The images of the gels collected from the gel documentation software Quantity One 1-D (Bio-Rad) were used for this analysis. The recommendations in the ImageJ User Guide for densitometry were followed, with some modifications, as described next. First, the images were adjusted to the 32-bit mode and the look-up table (LUT) was inverted. Then, the lanes of the gels containing the corresponding pDNA and RNA bands were selected using the Rectangular Selection tool. The profile plot representing the relative densities of the bands contained in the selected area was obtained using the Plot Lanes function of the Analyze Gels tool. The area of each peak, corresponding to each band, was measured with the help of the Wand tool. The Label Peaks function (Analyze Gels tool) was used to label each peak with its size, expressed as a percentage of the total size of all of the analysed peaks.

## 3. Results and Discussion

### 3.1. Specificity of PEG–Amino Affinity Ligands

PEG–dextran systems are among the most well-studied polymer–polymer two-phase systems. Their use for the separation and recovery of several biomolecules has been reported throughout the years. However, until now, there are no works reporting the application of this type of ATPS to efficiently separate pDNA molecules. This may be related to the subtle differences between the physicochemical properties of the two phases of polymer–polymer ATPSs, which makes them less suitable for separation applications [68]. Their low selectivity highly hampers the separation of pDNA molecules from complex mixtures, such as crude cell lysates.

Kepka and co-workers reported that, in PEG–dextran systems, all the main biomolecules that are present in *E. coli* lysate accumulate in the bottom phase [69]. Based on these findings, a PEG–dextran system was selected to test the purification of pDNA from crude cell lysates, based on an affinity partition approach. First, it was hypothesised that the PEG–amino affinity ligands would accumulate in the top PEG-rich phase of the PEG–dextran system due to physicochemical similarities with the top phase forming polymer PEG. Furthermore, the partition of the ligands to the top phase would be followed by the accumulation of the pDNA in the same phase.

**Figure 1:**
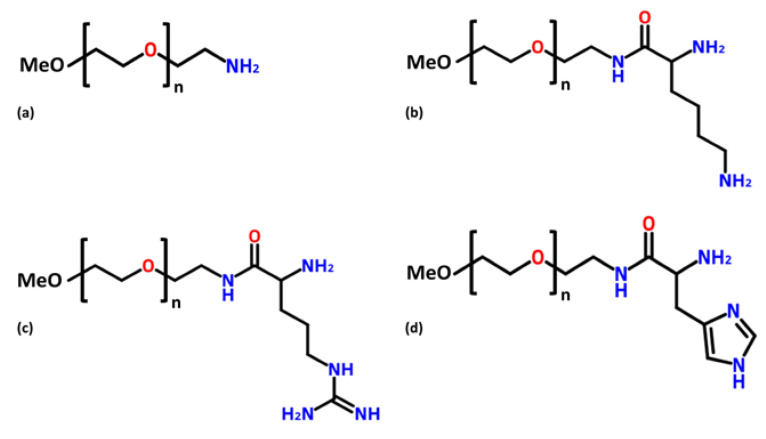
Poly(ethylene glycol)–amino ligands chemical structure: (**a**) PEG–amine; (**b**) PEG–lysine; (**c**) PEG–arginine, and (**d**) PEG–histidine.

The PEG–amino affinity ligands shown in Figure 1 were carefully chosen based on the results reported in previously reported affinity chromatography studies. In these studies, the capacity of positively charged amino groups to interact with the negatively charged pDNA molecules was demonstrated [59,70]. In the present study, the affinity ligands were obtained by derivatisation of PEG chains with amino groups (PEG–amine) or by conjugation with positively charged amino acids (PEG–lysine, PEG–arginine, and PEG–histidine). All ligands were positively charged due to the presence of the amino groups.

Considering the differences in the superficial charges of the ligands and the plasmid (negatively charged because of the phosphate groups), it was expected that the two species would interact electrostatically. This was certainly true for PEG–amine, but regarding the conjugated ligands, it is known that other types of interactions may occur. Although the mechanisms of pDNA biorecognition may differ between the affinity ligands, the result was expected to be the same. Since the ligands are based on PEG chains, they should accumulate in the top phase of the system (PEG-rich phase) and therefore be able to pull all pDNA molecules to this phase, keeping the remaining contaminants, mainly RNA and proteins, in the bottom dextran-rich phase.

**Figure 2:**
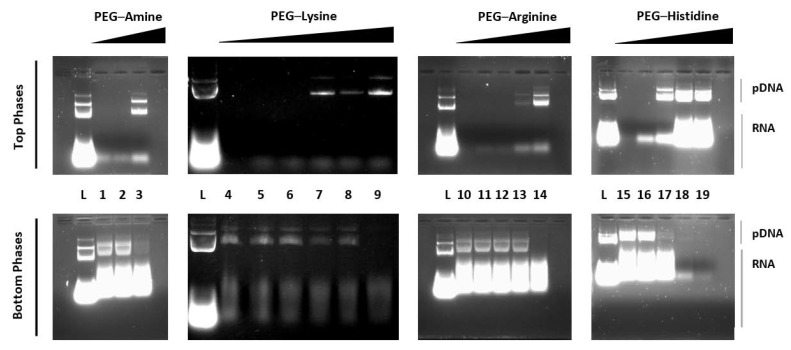
Agarose gel electrophoresis (AGE) analysis of nucleic acid partitioning in systems composed of 16.2% (*w*/*w*) PEG 600–17.4% (*w*/*w*) dextran 100 with 20% (*w*/*w*) of desalted bacterial lysate (pH = 7.5 or 8.5) and increasing concentrations of the amino affinity ligand. (L) Desalted lysate; (1–3) 6.3, 6.4, and 6.5% (*w*/*w*) PEG–amine; (4–9) 0, 1.0, 1.05, 1.1, 1.15, and 1.2% (*w*/*w*) PEG–lysine; (10–14) 0, 0.1, 0.2, 0.3, and 0.5% (*w*/*w*) PEG–arginine; (15–19) 0, 1, 1.3, 2, and 3% (*w*/*w*) PEG–histidine. All concentrations of the affinity ligand were relative to the 16.2% (*w*/*w*) PEG 600 total. The original AGE is shown in Figures S1–S4 of the Supplementary Materials.

Therefore, the first part of this work comprised a concentration screening for each ligand in the PEG–dextran systems to find the minimum ligand concentration that was needed to separate the pDNA from the other contaminants. Figure 2 shows the results for the pDNA and RNA partition in systems with increasing concentrations of PEG–amine, PEG–lysine, PEG–arginine, and PEG–histidine.

From the AGE analysis, it was clearly seen that, in the absence of an affinity ligand, all the nucleic acids accumulated in the bottom phase of the ATPSs. However, as the concentration of affinity ligand increased in the systems, pDNA molecules started to be steered to the top phases, verifying our initial hypothesis.

After the initial screening, the following minimum percentages of ligand were defined: 6.5% (*w*/*w*) PEG–amine, 1.2% (*w*/*w*) PEG–lysine, 0.5% (*w*/*w*) PEG–arginine, and 1.3% (*w*/*w*) PEG–histidine (note that the ligand percentage was related to the total percentage of PEG in the system).

Molecular dynamics models revealed that several amino acid–nucleotide interactions may occur [49,50]. The lysine lateral side chain length promotes interactions in different conformations, producing good geometries that allow for strong hydrogen bonds. Molecular modelling studies reported that, in protein–DNA interactions, lysine can promote complex hydrogen bonds preferentially with guanine [50]. Furthermore, lysine N atoms were found to be able to work both as acceptors and donors of hydrogen atoms [49].

Due to arginine’s property, namely, its ability to interact in different conformations, its side chain length and its tendency to form strong hydrogen bonds could be pointed to as the key mechanisms for the biorecognition of plasmids [49]. In fact, some studies reported the establishment of strong arginine-mediated interactions between proteins and nucleic acids [49,50]. Moreover, it was stated that the biorecognition of pDNA molecules could be due to many elementary interactions between the backbone of plasmids and/or involving the nitrogen bases [71]. It was also reported that the supercoiled structure of the pDNA molecules favours multiple contacts with the ligands and promotes more complex interactions between the two species [59].

Finally, regarding histidine, it was discussed by Sousa et al. [59] that this amino acid seems to interact with the exposed nitrogen bases of pDNA instead of its backbone. Several types of interactions seem to lead to histidine–pDNA binding, namely, hydrogen bonds, hydrophobic, imidazole ring- and water-mediated interactions, ring stacking, and van der Waals forces [49,50].

Even though the addition of the ligands led to an increase in the ATPSs’ selectivity towards pDNA, another phenomenon was identified. A residual amount of an unknown species of RNA was detected in the top phases of the systems along with pDNA. Remarkably, it was found that this RNA species was first steered to the top phase of the systems for lower ligand concentrations rather than the pDNA. These results suggest that even though the ligands showed an affinity for the pDNA, they seemed to have even more affinity to a certain type of RNA. A similar phenomenon was found in the affinity chromatography experiments using immobilised positively charged amino acids as affinity ligands. The authors reported higher relative retention factors for RNA than for pDNA for all the ligands except for arginine [55].

### 3.2. pDNA Extraction with an Ammonium-Sulphate-Rich Phase

The first evaluation of the affinity ligands showed that all of them could be used in a preliminary step of purification of pDNA or a multi-stage purification process, but not a single-step process. Thus, a second extraction was performed in an attempt to separate the pDNA from the remaining RNA.

PEG–ammonium sulphate ATPSs, previously studied by [40], were chosen based on the published results concerning their separation yields. Trindade and co-workers reported that a 20% (*w*/*w*) PEG 600–15% (*w*/*w*) ammonium sulphate system allows for the recovery of 80.6% of pDNA, protein, and RNA free in the bottom phase relative to the initial lysate (20% *w*/*w* load) [40].

**Figure 3:**
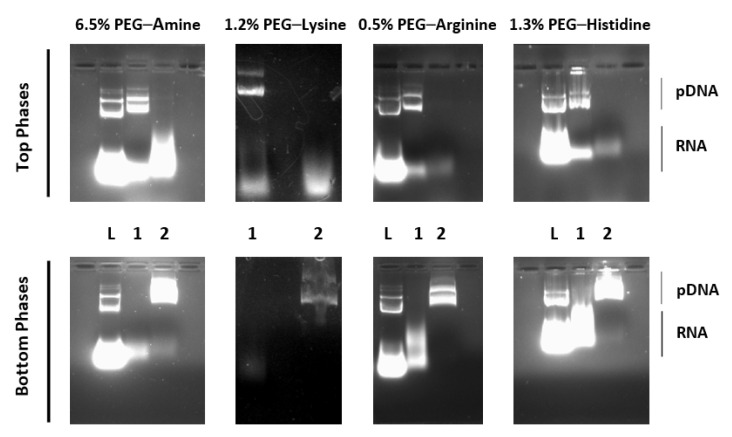
Agarose gel electrophoresis (AGE) analysis of nucleic acids partitioning in subsequent systems composed of (1) 16.2% (*w*/*w*) PEG 600–17.4% (*w*/*w*) dextran 100 with 20% (*w*/*w*) of desalted bacterial lysate (pH = 7.5 or 8.5) with the corresponding affinity ligand percentage and (2) 20% (*w*/*w*) PEG 600–15% (*w*/*w*) ammonium sulphate systems. (L) Desalted lysate. The original AGE is shown in Figures S5–S8 of the Supplementary Materials.

Therefore, a second extraction with a bottom ammonium-sulphate-rich phase was tested to verify whether the pDNA partition could be shifted. In this new system, it was expected that the accumulation of pDNA would be in the bottom phase, while the remaining contaminants would partition to the upper phase. The results of these experiments are shown in Figure 3, where samples of each phase of the first and the second ATPS were analysed using AGE.

In all cases, a significant plasmid–RNA separation was achieved after performing the second extraction. Despite RNA being completely removed in the systems containing PEG–lysine and PEG–arginine, it was still possible to see a vestigial amount of it in the bottom phases of the second systems (where pDNA accumulates) when PEG–amine and PEG–histidine were used. Similarities between the molecular weight and the net charge of some RNA molecules or fragments and pDNA may explain why, in these systems, the former ones were more suitable to partition to the salt-rich phase. Moreover, even though the second system was prepared to have a phase volume ratio of 1.1, higher ratios were observed. As the volume of the bottom phase of the second system has drastically decreased, the partitioning phenomenon may have occurred differently, affecting the distribution of the biomolecules [72].

Table 1 shows the quantification results of the AGE bands corresponding to the partition of pDNA and RNA after re-extraction with the second system. The results obtained after the densitometric analysis of the agarose gels with the image processing software corroborated the conclusions drawn before. The software could neither detect nor quantify any bands corresponding to RNA in the bottom phases of the systems where PEG–lysine and PEG–arginine were added.

### 3.3. Protein Contamination Assessment

**Figure 4:**
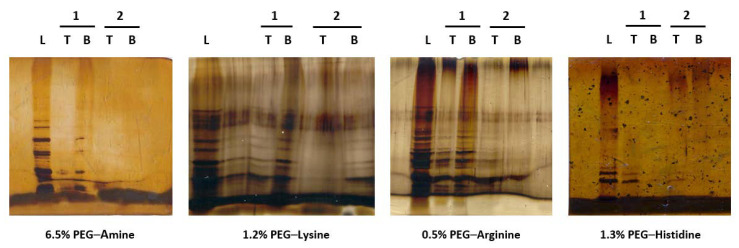
SDS–PAGE electrophoresis analysis of the total proteins partitioning in subsequent systems composed by (1) 16.2% (*w*/*w*) PEG 600–17.4% (*w*/*w*) dextran 100 with 20% (*w*/*w*) desalted bacterial lysate (pH = 7.5 or 8.5) with the corresponding affinity ligand percentage and (2) 20% (*w*/*w*) PEG 600–15% (*w*/*w*) ammonium sulphate systems. (L) Desalted lysate. The original SDS–PAGE is shown in Figures S9–S12 of the Supplementary Materials.

Concerning the protein fraction that was present in the cell lysate (170.09 µg·mL^−1^ in non-desalted lysates and 104.66 µg·mL^−1^ in the desalted lysates; total protein content quantified using the Bradford Method [65,73]), its partition behaviour was assessed using SDS–PAGE (Figure 4).

It was possible to conclude that, after the first partition experiment, most of the proteins were accumulated in the bottom phase of the systems. This meant that, after the first step of the purification, the proteins were almost completely removed. In the second system, the remaining proteins were retained in the top phase, leaving the pDNA (collected in the bottom phase) free of its contamination.

### 3.4. Process Scale-Up and Capacity Assessment

**Figure 5:**
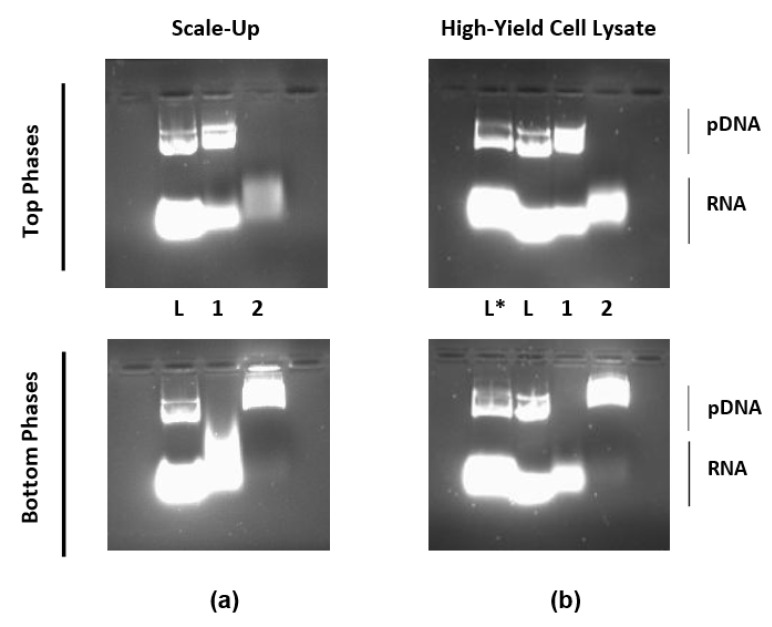
Agarose gel electrophoresis (AGE) analysis of nucleic acids partitioning in subsequent systems composed by (1) 16.2% (*w*/*w*) PEG 600–17.4% (*w*/*w*) dextran 100 with 20% (*w*/*w*) of desalted bacterial lysate, pH = 7.5, and with 0.5% (*w*/*w*) PEG–arginine; and (2) 20% (*w*/*w*) PEG 600–15% (*w*/*w*) ammonium sulphate systems. (L) Desalted lysate; (L*) non-desalted lysate; (**a**) samples from the scale-up experiments; (**b**) samples from experiments with high-yield pDNA cell lysate. The original AGE is shown in Figures S13 and S14 of the Supplementary Materials.

Once the biorecognition capacity of each amino affinity ligand was assessed, PEG–arginine was selected for further tests. To verify whether the purification outcome of this two-step process was the same in a larger system, the scale of the systems was increased between 2 and 20 times. Figure 5a shows the AGE results for the biggest system tested (10 g). The results from the scale-up experiment confirmed that there were no changes in the partition behaviour of the pDNA molecules during the process. These findings corroborate the well-known theoretical concepts around ATPSs as a separation technique, which state that this methodology is easily scaled up. Furthermore, this experiment can be used as a proof of concept of the affinity approach for plasmid purification from crude cell lysates.

Additionally, to assess the capacity of the systems, a new pDNA-concentrated cell lysate was used. This new lysate was obtained from the alkaline lysis of a genetically modified *E. coli* strain, namely, DH5α (ackA-pta) (poxB), which is known to be a high pDNA producer. The results, represented in Figure 5b, expressed the high capacity of the systems under study since the same results for biomolecules’ partition were obtained. Once again, the well-known high capacity of ATPSs was shown with this experiment.

However, it is important to mention that a vestigial amount of RNA contaminating the pDNA-containing bottom phase of the second system was seen in both tests. This suggests that the concentration of the ligand might have to be optimised depending on the scale and pDNA concentration. Alternatively, a final polishing step might have to be introduced to attain a higher degree of purification, particularly in larger-scale applications.

## 4. Conclusions

In this work, it was demonstrated that positively charged amino acids that were conjugated to PEG chains could be used as affinity ligands for the purification of pDNA in ATPSs. Moreover, the commercial polymer PEG–amine was shown to have the capacity to biorecognise the molecules of pDNA. However, the interaction of these ligands with the pDNA was different for each ligand, which was reflected in the differences in the amounts of ligands needed.

Regarding the application of amino acids for pDNA purification in affinity chromatography, it was reported that the performances of the lysine and histidine were very similar (recovery yield of 45%) and that arginine was the best ligand (recovery yield of 79%) [55]. Compared to the results obtained in this study, it is possible to see that the same tendency can be seen in ATPSs. The amount of PEG–lysine and PEG–histidine was almost the same and the best performance was reached with PEG–arginine.

Furthermore, even though the affinity of the ligands to a particular type of RNA seemed to be higher than for the pDNA itself, this was mostly overcome with the second extraction. Extraction of the pDNA from the PEG-rich phase to an ammonium sulphate phase yielded purified pDNA preparations without protein contamination and with a residual presence of RNA. Since the final plasmid solutions had a considerable salt concentration, their final recovery and concentration could be successfully achieved via centrifuge filtration using Amicon Ultra-15 Centrifugal Filter Units (data not shown).

Finally, it was demonstrated that this affinity purification approach was suitable to be implemented at a larger scale and could be used to purify pDNA from highly concentrated crude cell lysates. In addition, the high affinity that was observed for a certain type of RNA (not yet identified) opens perspectives for the utilisation of these ligands in the purification of this biomolecule for RNA therapeutics, such as noncoding RNAs and mRNA [74,75].

## Figures and Tables

**Table 1:** Characterisation of the nucleic acids band intensities after the AGE analysis of the partitioning of pDNA and RNA with the re-extraction system (20% (*w*/*w*) PEG 600–15% (*w*/*w*) ammonium sulphate).

	**6.5% PEG–Amine**			
	**Top Phase**		**Bottom Phase**	
	**Band Area**	**Percentage**	**Band Area**	**Percentage**
**pDNA**	4525.3	5.1	58,908.5	74.1
**RNA**	84,927.4	94.9	20,622.5	25.9
	**1.2% PEG–Lysine**			
	**Top Phase**		**Bottom Phase**	
	**Band Area**	**Percentage**	**Band Area**	**Percentage**
**pDNA**	0	0	50,694.5	100
**RNA**	43254.3	100	0	0
	**0.5% PEG–Arginine**			
	**Top Phase**		**Bottom Phase**	
	**Band Area**	**Percentage**	**Band Area**	**Percentage**
**pDNA**	0	0	48,787.4	100
**RNA**	54,061.7	100	0	0
	**1.3% PEG–Histidine**			
	**Top Phase**		**Bottom Phase**	
	**Band Area**	**Percentage**	**Band Area**	**Percentage**
**pDNA**	0	0	47,470.7	81.3
**RNA**	30,013.5	100	10,904.5	18.7
	**0.5% PEG–Arginine (Scale-Up)**			
	**Top Phase**		**Bottom Phase**	
	**Band Area**	**Percentage**	**Band Area**	**Percentage**
**pDNA**	0	0	29,553.5	77.5
**RNA**	39,394.4	100	8601.0	22.5
	**0.5% PEG–Arginine (High-Yield Cell Lysate)**			
	**Top Phase**		**Bottom Phase**	
	**Band Area**	**Percentage**	**Band Area**	**Percentage**
**pDNA**	0	0	24,504.4	81.8
**RNA**	28,249.9	100	5453.5	18.2

## Data Availability

All datasets generated for this study are included in the article.

## Supplementary Materials

The following are available online at https://www.mdpi.com/article/10.3390/life11111138/s1, Figure S1: Original Agarose gel electrophoresis (AGE) shown in Figure 2, lanes 1–3: analysis of nucleic acids partitioning in systems composed by 16.2% (*w*/*w*) PEG 600–17.4% (*w*/*w*) dextran 100 with 20% (*w*/*w*) of desalted bacterial lysate (pH = 7.5 or 8.5) and crescent concentrations of PEG–amine. (L) Desalted lysate; (1–3) 6.3, 6.4 and 6.5% (*w*/*w*) PEG–amine. All concentrations of affinity ligand are relative to the 16.2% (*w*/*w*) total PEG 600., Figure S2: Original Agarose gel electrophoresis (AGE) shown in Figure 2, lanes 4–9: analysis of nucleic acids partitioning in systems composed by 16.2% (*w*/*w*) PEG 600–17.4% (*w*/*w*) dextran 100 with 20% (*w*/*w*) of desalted bacterial lysate (pH = 7.5 or 8.5) and crescent concentrations of PEG–lysine. (PP) Pure pDNA; (L) Desalted lysate; (4–9) 0, 1.0, 1.05, 1.1, 1.15 and 1.2% (w/w) PEG–lysine. All concentrations of affinity ligand are relative to the 16.2% (*w*/*w*) total PEG 600., Figure S3: Original Agarose gel electrophoresis (AGE) shown in Figure 2, lanes 10–14: analysis of nucleic acids partitioning in systems composed by 16.2% (*w*/*w*) PEG 600–17.4% (*w*/*w*) dextran 100 with 20% (*w*/*w*) of desalted bacterial lysate (pH = 7.5 or 8.5) and crescent concentrations of PEG–arginine. (L) Desalted lysate; (10–14) 0, 0.1, 0.2, 0.3 and 0.5% (*w*/*w*) PEG–arginine. All concentrations of affinity ligand are relative to the 16.2% (*w*/*w*) total PEG 600., Figure S4: Original Agarose gel electrophoresis (AGE) shown in Figure 2, lanes 15–19: analysis of nucleic acids partitioning in systems composed by 16.2% (*w*/*w*) PEG 600–17.4% (*w*/*w*) dextran 100 with 20% (*w*/*w*) of desalted bacterial lysate (pH = 7.5 or 8.5) and crescent concentrations of PEG–histidine. (L) Desalted lysate; (15–19) 0, 1, 1.3, 2 and 3% (*w*/*w*) PEG–histidine. All concentrations of affinity ligand are relative to the 16.2% (*w*/*w*) total PEG 600., Figure S5: Original Agarose gel electrophoresis (AGE) shown in Figure 3: analysis of nucleic acids partitioning in systems composed by (1) 16.2% (*w*/*w*) PEG 600–17.4% (*w*/*w*) dextran 100 with 20% (*w*/*w*) of desalted bacterial lysate (pH = 7.5 or 8.5) with 6.5% PEG–amine and (2) 20% (*w*/*w*) PEG 600–15% (*w*/*w*) ammonium sulphate systems. (L) Desalted lysate., Figure S6: Original Agarose gel electrophoresis (AGE) shown in Figure 3: analysis of nucleic acids partitioning in systems composed by (1) 16.2% (*w*/*w*) PEG 600–17.4% (*w*/*w*) dextran 100 with 20% (*w*/*w*) of desalted bacterial lysate (pH = 7.5 or 8.5) with 1.2% PEG–lysine and (2) 20% (*w*/*w*) PEG 600–15% (*w*/*w*) ammonium sulphate systems. (PP) pure pDNA; (L) Desalted lysate., Figure S7: Original Agarose gel electrophoresis (AGE) shown in Figure 3: analysis of nucleic acids partitioning in systems composed by (1) 16.2% (*w*/*w*) PEG 600–17.4% (*w*/*w*) dextran 100 with 20% (*w*/*w*) of desalted bacterial lysate (pH = 7.5 or 8.5) with 0.5% PEG–arginine and (2) 20% (*w*/*w*) PEG 600–15% (*w*/*w*) ammonium sulphate systems. (L) Desalted lysate., Figure S8: Original Agarose gel electrophoresis (AGE) shown in Figure 3: analysis of nucleic acids partitioning in systems composed by (1) 16.2% (*w*/*w*) PEG 600–17.4% (*w*/*w*) dextran 100 with 20% (*w*/*w*) of desalted bacterial lysate (pH = 7.5 or 8.5) with 1.3% PEG–histidine and (2) 20% (*w*/*w*) PEG 600–15% (*w*/*w*) ammonium sulphate systems. (L) Desalted lysate., Figure S9: Original SDS-PAGE electrophoresis shown in Figure 4: analysis of total proteins partitioning in systems composed by (1) 16.2% (*w*/*w*) PEG 600–17.4% (*w*/*w*) dextran 100 with 20% (*w*/*w*) of desalted bacterial lysate (pH = 7.5 or 8.5) with 6.5% PEG–amine and (2) 20% (*w*/*w*) PEG 600–15% (*w*/*w*) ammonium sulphate systems. (L) Desalted lysate., Figure S10: Original SDS-PAGE electrophoresis shown in Figure 4: analysis of total proteins partitioning in systems composed by (1) 16.2% (*w*/*w*) PEG 600–17.4% (*w*/*w*) dextran 100 with 20% (*w*/*w*) of desalted bacterial lysate (pH = 7.5 or 8.5) with 1.2% PEG–lysine and (2) 20% (*w*/*w*) PEG 600–15% (*w*/*w*) ammonium sulphate systems. (L) Desalted lysate., Figure S11: Original SDS-PAGE electrophoresis shown in Figure 4: analysis of total proteins partitioning in systems composed by (1) 16.2% (*w*/*w*) PEG 600–17.4% (*w*/*w*) dextran 100 with 20% (*w*/*w*) of desalted bacterial lysate (pH = 7.5 or 8.5) with 0.5% PEG–arginine and (2) 20% (*w*/*w*) PEG 600–15% (*w*/*w*) ammonium sulphate systems. (L) Desalted lysate., Figure S12: Original SDS-PAGE electrophoresis shown in Figure 4: analysis of total proteins partitioning in systems composed by (1) 16.2% (*w*/*w*) PEG 600–17.4% (*w*/*w*) dextran 100 with 20% (*w*/*w*) of desalted bacterial lysate (pH = 7.5 or 8.5) with 1.3% PEG–histidine and (2) 20% (*w*/*w*) PEG 600–15% (*w*/*w*) ammonium sulphate systems. (L) Desalted lysate., Figure S13: Original Agarose gel electrophoresis (AGE) shown in Figure 5(a): analysis of nucleic acids partitioning in the scale-up experiments of systems composed by (1) 16.2% (*w*/*w*) PEG 600–17.4% (*w*/*w*) dextran 100 with 20% (*w*/*w*) of desalted bacterial lysate (pH = 7.5 or 8.5) with 0.5% PEG–arginine and (2) 20% (*w*/*w*) PEG 600–15% (*w*/*w*) ammonium sulphate systems. (L) Desalted lysate., Figure S14: Original Agarose gel electrophoresis (AGE) shown in Figure 5(b): analysis of nucleic acids partitioning in the experiments of systems composed by (1) 16.2% (*w*/*w*) PEG 600–17.4% (*w*/*w*) dextran 100 with 20% (*w*/*w*) of desalted bacterial lysate with high pDNA production yield (pH = 7.5 or 8.5) with 0.5% PEG–arginine and (2) 20% (*w*/*w*) PEG 600–15% (*w*/*w*) ammonium sulphate systems. (L*) Non-desalted lysate; (L) Desalted lysate.
