# Supplementary material for: Initial Screening of Poly(ethylene glycol) Amino Ligands for Affinity Purification of Plasmid DNA in Aqueous Two-Phase Systems
Source: Life (Basel). 2021 Oct 26;11(11):1138. doi: 10.3390/life11111138 (PMC8619368; doi:10.3390/life11111138)
Supplement: Supplementary file 1 [file life-11-01138-s001.zip › life-1414902-supplementary.pdf]

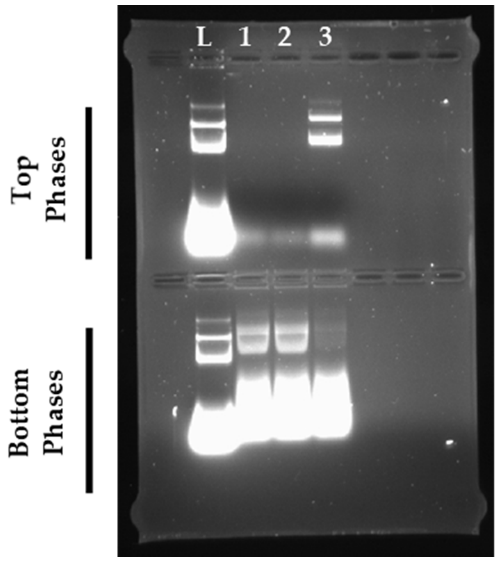

Figure S1. Original Agarose gel electrophoresis (AGE) shown in Figure 2, lanes 1-3: analysis of nucleic acids partitioning in systems composed by 16.2% (w/w) PEG 600 – 17.4% (w/w) dextran 100 with 20% (w/w) of desalted bacterial lysate (pH = 7.5 or 8.5) and crescent concentrations of PEG-amine. (L) Desalted lysate; (1-3) 6.3, 6.4 and 6.5% (w/w) PEG-amine. All concentrations of affinity ligand are relative to the 16.2% (w/w) total PEG 600.

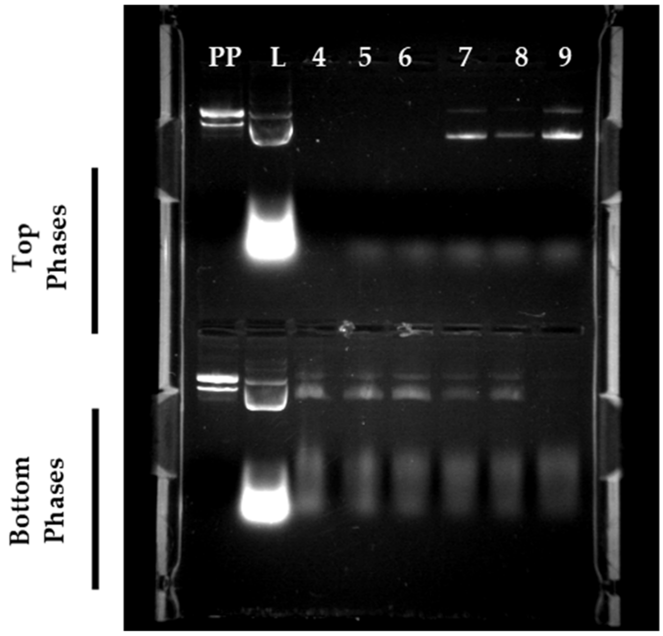

Figure S2. Original Agarose gel electrophoresis (AGE) shown in Figure 2, lanes 4-9: analysis of nucleic acids partitioning in systems composed by 16.2% (w/w) PEG 600 – 17.4% (w/w) dextran 100 with 20% (w/w) of desalted bacterial lysate (pH = 7.5 or 8.5) and crescent concentrations of PEG-Lysine. (PP) Pure pDNA; (L) Desalted lysate; (4-9) 0, 1.0, 1.05, 1.1, 1.15 and 1.2% (w/w) PEG-Lysine. All concentrations of affinity ligand are relative to the 16.2% (w/w) total PEG 600.

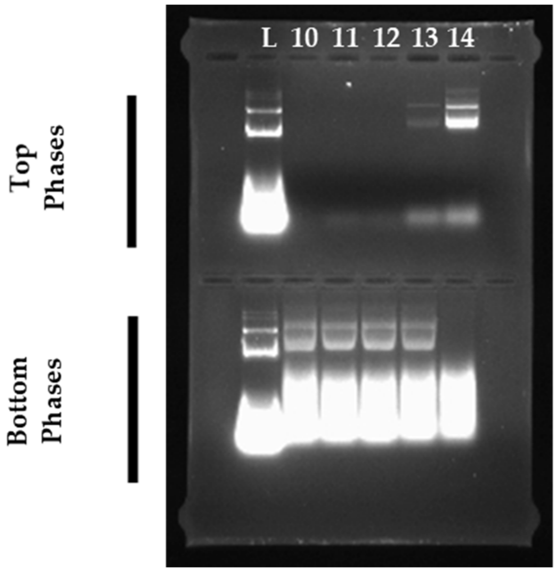

Figure S3. (10-14). Original Agarose gel electrophoresis (AGE) shown in Figure 2, lanes 10-14: analysis of nucleic acids partitioning in systems composed by 16.2% (w/w) PEG 600 – 17.4% (w/w) dextran 100 with 20% (w/w) of desalted bacterial lysate (pH = 7.5 or 8.5) and crescent concentrations of PEG-Arginine. (L) Desalted lysate; (10-14) 0, 0.1, 0.2, 0.3 and 0.5% (w/w) PEG-Arginine. All concentrations of affinity ligand are relative to the 16.2% (w/w) total PEG 600.

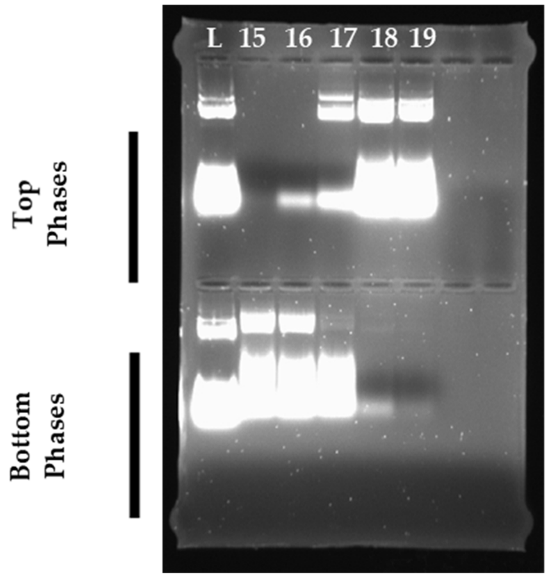

Figure S4. Original Agarose gel electrophoresis (AGE) shown in Figure 2, lanes 15-19: analysis of nucleic acids partitioning in systems composed by 16.2% (w/w) PEG 600 – 17.4% (w/w) dextran 100 with 20% (w/w) of desalted bacterial lysate (pH = 7.5 or 8.5) and crescent concentrations of PEG-Histidine. (L) Desalted lysate; (15-19) 0, 1, 1.3, 2 and 3% (w/w) PEG-Histidine. All concentrations of affinity ligand are relative to the 16.2% (w/w) total PEG 600.

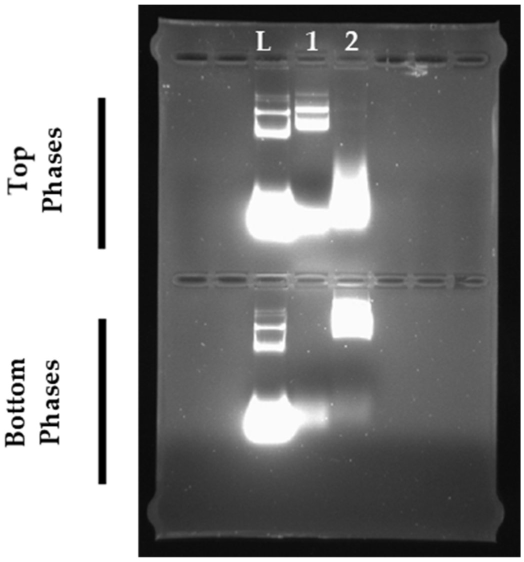

Figure S5. Original Agarose gel electrophoresis (AGE) shown in Figure 3: analysis of nucleic acids partitioning in systems composed by (1) 16.2% (w/w) PEG 600 – 17.4% (w/w) dextran 100 with 20% (w/w) of desalted bacterial lysate (pH = 7.5 or 8.5) with 6.5% PEG-amine and (2) 20% (w/w) PEG 600 – 15% (w/w) ammonium sulphate systems. (L) Desalted lysate.

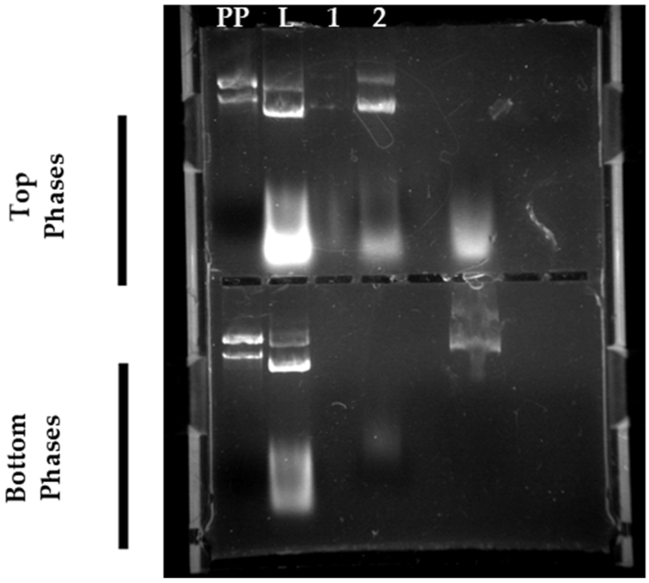

Figure S6. Original Agarose gel electrophoresis (AGE) shown in Figure 3: analysis of nucleic acids partitioning in systems composed by (1) 16.2% (w/w) PEG 600 – 17.4% (w/w) dextran 100 with 20% (w/w) of desalted bacterial lysate (pH = 7.5 or 8.5) with 1.2% PEG-Lysine and (2) 20% (w/w) PEG 600 – 15% (w/w) ammonium sulphate systems. (PP) pure pDNA; (L) Desalted lysate.

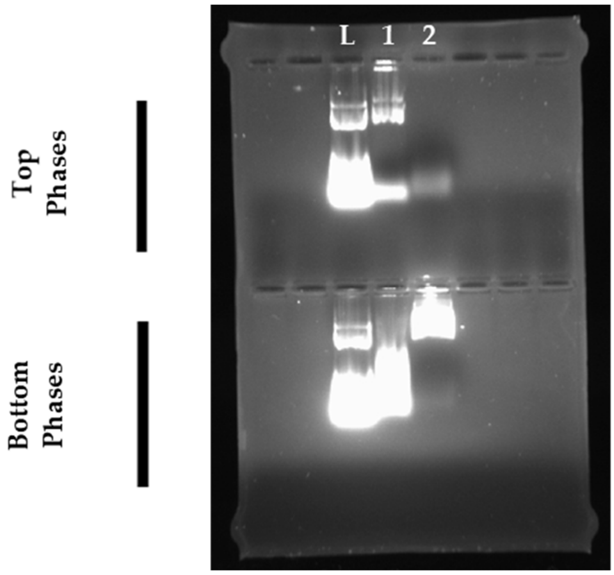

Figure S7. Original Agarose gel electrophoresis (AGE) shown in Figure 3: analysis of nucleic acids partitioning in systems composed by (1) 16.2% (w/w) PEG 600 – 17.4% (w/w) dextran 100 with 20% (w/w) of desalted bacterial lysate (pH = 7.5 or 8.5) with 0.5% PEG-Arginine and (2) 20% (w/w) PEG 600 – 15% (w/w) ammonium sulphate systems. (L) Desalted lysate.

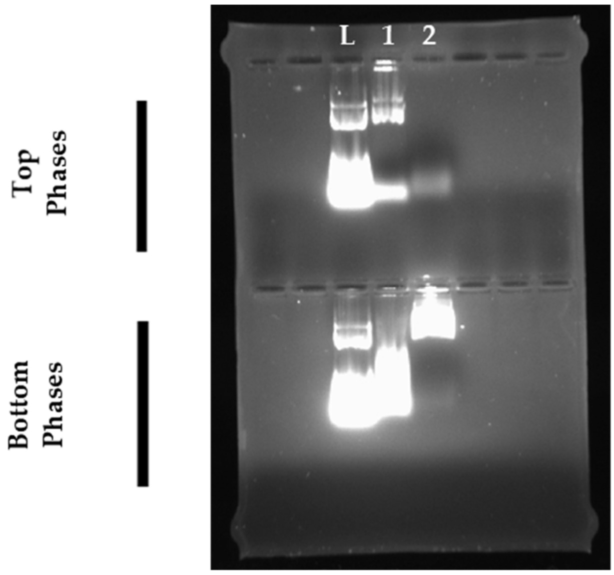

Figure S8. Original Agarose gel electrophoresis (AGE) shown in Figure 3: analysis of nucleic acids partitioning in systems composed by (1) 16.2% (w/w) PEG 600 – 17.4% (w/w) dextran 100 with 20% (w/w) of desalted bacterial lysate (pH = 7.5 or 8.5) with 1.3% PEG-Histidine and (2) 20% (w/w) PEG 600 – 15% (w/w) ammonium sulphate systems. (L) Desalted lysate.

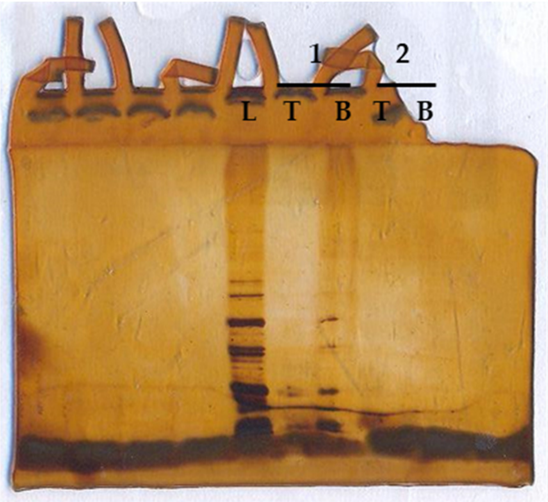

Figure S9. Original SDS-PAGE electrophoresis shown in Figure 4: analysis of total proteins partitioning in systems composed by (1) 16.2% (w/w) PEG 600 – 17.4% (w/w) dextran 100 with 20% (w/w) of desalted bacterial lysate (pH = 7.5 or 8.5) with 6.5% PEG-amine and (2) 20% (w/w) PEG 600 – 15% (w/w) ammonium sulphate systems. (L) Desalted lysate.

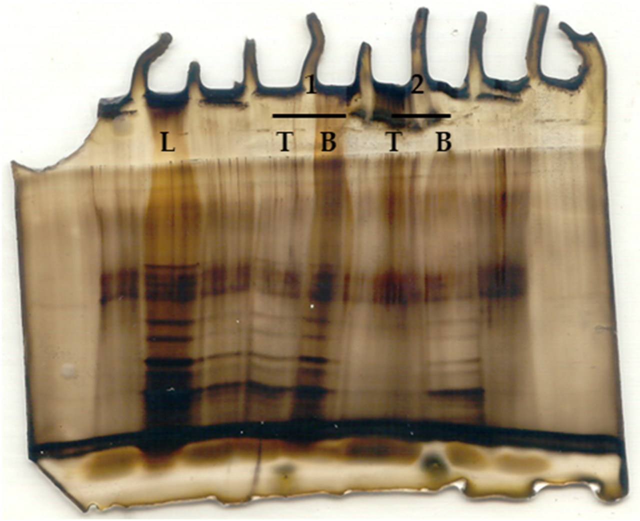

Figure S10. Original SDS-PAGE electrophoresis shown in Figure 4: analysis of total proteins partitioning in systems composed by (1) 16.2% (w/w) PEG 600 – 17.4% (w/w) dextran 100 with 20% (w/w) of desalted bacterial lysate (pH = 7.5 or 8.5) with 1.2% PEG-Lysine and (2) 20% (w/w) PEG 600 – 15% (w/w) ammonium sulphate systems. (L) Desalted lysate.

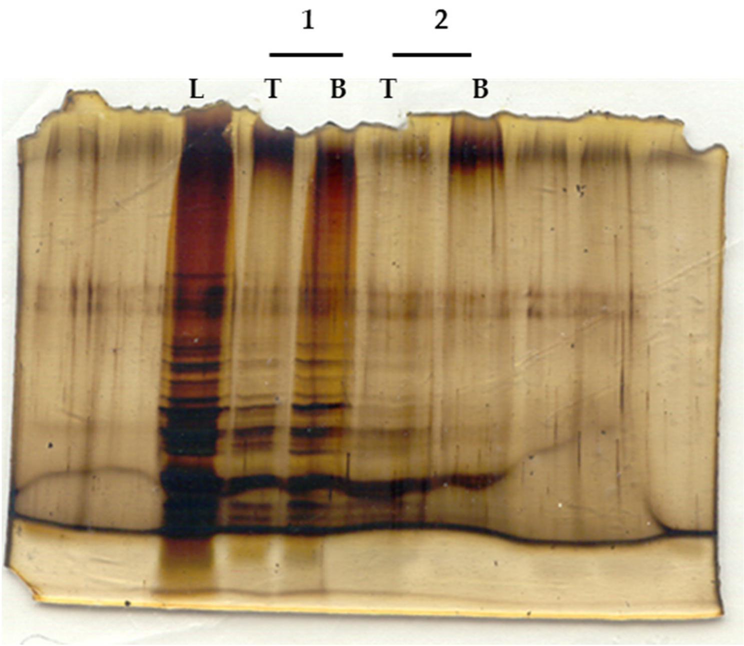

Figure S11. Original SDS-PAGE electrophoresis shown in Figure 4: analysis of total proteins partitioning in systems composed by (1) 16.2% (w/w) PEG 600 – 17.4% (w/w) dextran 100 with 20% (w/w) of desalted bacterial lysate (pH = 7.5 or 8.5) with 0.5% PEG-Arginine and (2) 20% (w/w) PEG 600 – 15% (w/w) ammonium sulphate systems. (L) Desalted lysate.

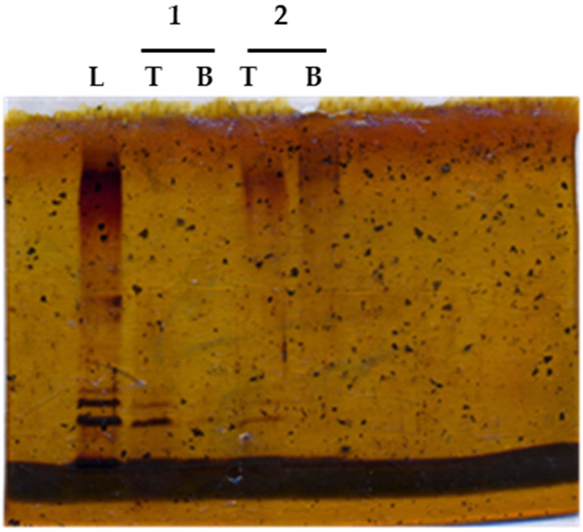

Figure S12. Original SDS-PAGE electrophoresis shown in Figure 4: analysis of total proteins partitioning in systems composed by (1) 16.2% (w/w) PEG 600 – 17.4% (w/w) dextran 100 with 20% (w/w) of desalted bacterial lysate (pH = 7.5 or 8.5) with 1.3% PEG-Histidine and (2) 20% (w/w) PEG 600 – 15% (w/w) ammonium sulphate systems. (L) Desalted lysate.

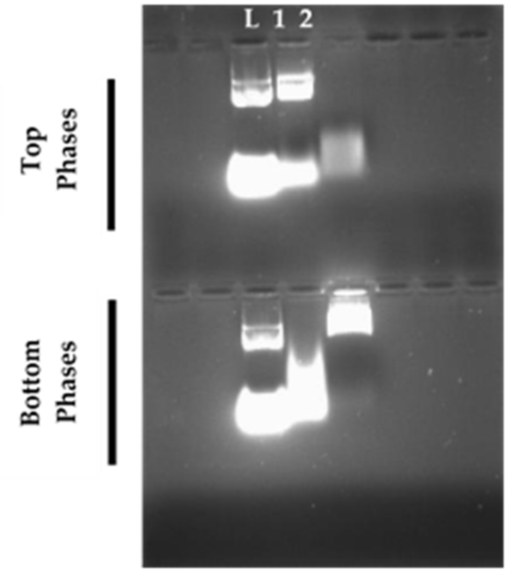

Figure S13. Original Agarose gel electrophoresis (AGE) shown in Figure 5(a): analysis of nucleic acids partitioning in the scale-up experiments of systems composed by (1) 16.2% (w/w) PEG 600 – 17.4% (w/w) dextran 100 with 20% (w/w) of desalted bacterial lysate (pH = 7.5 or 8.5) with 0.5% PEG-Arginine and (2) 20% (w/w) PEG 600 – 15% (w/w) ammonium sulphate systems. (L) Desalted lysate.

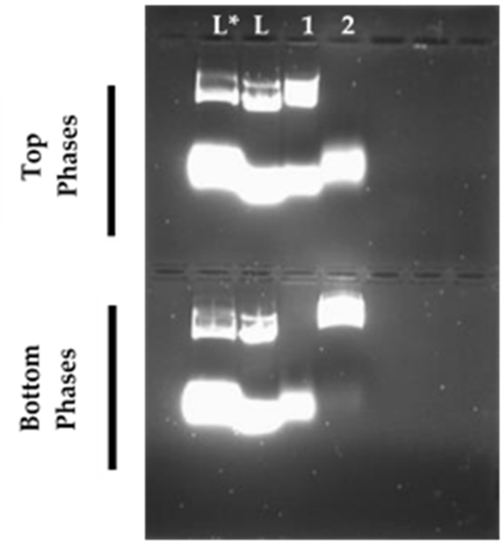

Figure S14. Original Agarose gel electrophoresis (AGE) shown in Figure 5(b): analysis of nucleic acids partitioning in the experiments of systems composed by (1) 16.2% (w/w) PEG 600 – 17.4% (w/w) dextran 100 with 20% (w/w) of desalted bacterial lysate with high pDNA production yield (pH = 7.5 or 8.5) with 0.5% PEG-Arginine and (2) 20% (w/w) PEG 600 – 15% (w/w) ammonium sulphate systems. (L*) Non-desalted lysate; (L) Desalted lysate.
